# Prevalence of dengue virus serotypes in the 2017 outbreak in Peshawar, KP, Pakistan

**DOI:** 10.1002/jcla.23371

**Published:** 2020-07-22

**Authors:** Najeeb Ullah Khan, Lubna Danish, Hydayat Ullah Khan, Maryam Shah, Muhammad Ismail, Ijaz Ali, Arnolfo Petruzziello, Rocco Sabatino, Annunziata Guzzo, Gerardo Botti, Aqib Iqbal

**Affiliations:** ^1^ Institute of Biotechnology and Genetic Engineering (Health Division) University of Agriculture Peshawar Pakistan; ^2^ Sulaiman Bin Abdullah Aba Al‐Khail Centre for Interdisciplinary in Basic Sciences (SA‐CIRBS) International Islamic University Islamabad Pakistan; ^3^ Peshawar PCR Lab Peshawar Pakistan; ^4^ Department of Zoology Islamia College University Peshawar Pakistan; ^5^ Department of Biosciences COMSATs University Islamabad Islamabad Pakistan; ^6^ UOC Clinical Pathology AORN Sant’Anna e San Sebastiano Caserta Italy; ^7^ Unit of Molecular Biology and Viral Oncology Istituto Nazionale Tumori ‐ IRCCS Fondazione “G. Pascale” Naples Italy; ^8^ SSD Transfusion Medicine Istituto Nazionale Tumori – IRCCS Fondazione “G. Pascale” Naples Italy; ^9^ Scientific Direction Istituto Nazionale Tumori ‐ IRCCS Fondazione “G. Pascale” Naples Italy

**Keywords:** dengue, dengue virus, KP‐Pakistan, prevalence, serotyping

## Abstract

**Background:**

Dengue is a viral disease, transmitted by infected *Aedes aegypti* and *Aedes albopictu*s female mosquitoes. Worldwide, 96 million infections were estimated in 2010. The dengue virus comprises four distinct serotypes (DENV‐1, DENV‐2, DENV‐3, and DENV‐4) which belong to the genus Flavivirus. Determining the serotypes during dengue outbreaks is crucial for its effective management in terms of diagnostics improvement and polyvalent vaccine development. The aim of the present study is to determine the prevalence rate of dengue virus serotypes in the samples collected from patients during the 2017 outbreak in Khyber Pakhtunkhwa, Pakistan.

**Methods:**

A total of 800 ELISA‐positive samples were collected, of which 513 (290 males, 223 females) samples were confirmed positive by PCR.

**Results:**

Out of 513, 25 were found serotype 1 (5%), 196 were serotype 2 (38%), 192 were serotype 3 (37%), 56 were serotype 4 (11%), and 44 (8%) were found to have mix serotypes.

**Conclusion:**

We can conclude that serotypes 2 and 3 of dengue virus were the predominated serotypes of dengue virus in the 2017 outbreak in Peshawar, capital city of Khyber Pakhtunkhwa, Pakistan.

## 1 INTRODUCTION

Dengue is mosquito‐borne viral infection, which affects about 390 million people every year.^1^ In the last decade, 120 countries are facing great challenge to prevent dengue virus (DENV) transmission and 2.5 billion world populations are at risk of dengue infection according to WHO report.^3^ During the dengue outbreaks in Pakistan, more than 55 000 individuals were hospitalized and many of them are children. Since 2006, dengue epidemics have occurred every year and have become a major health problem in Pakistan.^2^

Dengue is endemic in most tropical and subtropical countries,^4^ and most of the dengue spread has caused by large number of human migration. *Aedes aegypti* mosquito is native to Africa but worldwide migration caused the spread of dengue, and Ae. Aegypti became one of major vector of dengue spread.^4^ The epidemiology and spread of DENV became a serious burden in South East Asia after Second World War.^5^ The first recorded epidemic of dengue hemorrhagic fever (DHF) was recorded in Philippines (Manila) in 1953‐1954, followed by second emergence in 1956,^5^ in Bangkok in 1958, and in Thailand in 1960s.^5^, ^6^ Until 1970s, only nine countries reported the presence of DHF which increased in 1995.^7^

The first case of dengue in Pakistan was reported from Hub, in Baluchistan in 1960, at that time Pakistan population was 45.9 million. From 1960 to 1980, there were only 12 dengue cases reported in Pakistan.^8^ In Pakistan, the first outbreak of DENV was detected in 1994, in which two distinct serotypes DENV‐2 and DENV‐3 had been identified in Karachi.^9^

Dengue virus is a positive single‐stranded RNA virus composed of about 11 kilobase (kb) that codes for 3411 amino acids polypeptide molecule. The polypeptide gives rise to structural proteins (membrane, envelope, and capsid) which are responsible for the basic structure of virus and are not take part in the viral genome replication and to non‐structural proteins (NS5, NS4B, NS4A, NS3, NS2B, NS2A, NS1) which are expressed inside the infected cells.^10^ Globally, the variants of dengue virus have been classified into four serotypes named DENV‐1, DENV‐2, DENV‐3, and DENV‐4, belonging to family named “Flaviviridae” of “Flavivirus” genus. The four serotypes of dengue virus have been originated from sylvatic strains, found in South East Asia forests.^11^ Each serotype of DENV consists of different genetic composition and undergoes genetic mutation that makes difficult to produce vaccines against all four serotypes. If patients gain immunity against one serotype, then immunity will be maintained against that serotype, but immunity against another serotype would be temporary.^12^

Dengue virus infection can cause a wide spectrum of clinical manifestations, which differ from dengue fever (DF) and severe dengue disease (SDD), which includes dengue hemorrhagic fever (DHF) and dengue shock syndrome (DSS).^2^

Dengue fever (DF) is characterized with high fever onset. In young children, neurological disturbances caused by dehydration may occur during febrile phase.^13^ DF can cause extreme weakness along with myalgia, leucopenia, petechiae rash, arthralgia, and eye pain.^14^ DF is also called breakbone fever due to severe pain in muscles and joints. There is no difference between the symptoms of DF and DHF during early stages. Although DHF leads to manifestations of viral hemorrhagic, vascular permeability that causes plasma leakage and thrombocytopenia.^15^ On the other hand, dengue shock syndrome (DSS) is different from DHF because it causes plasma leakage, a process in which the protein‐rich fluid component of the blood leaks from blood vessels into interstitial spaces and leads to shock.^16^ Plasma leakage is the major cause of mortality and morbidity in patients with DSS.

The conditions of about 5%‐10% of patients with DF can become severe; hospitalization is required for the patients suffering from DHF. About 30%‐40% of children suffering from DHF can contract DSS. Treatment is symptomatic and supportive. Fatality rate due to DSS/DHF can reach 5%. The patients recovered from DF acquired immunity for the life time, against the serotype of dengue that had caused infection in the patient.^17^

Determining the dengue serotypes during a particular outbreak of the disease is very important not only for its effective management but above all for improving diagnostics and for the development of polyvalent vaccine. Considering the current scenario of dengue infection in Pakistan, it is of prime importance to investigate the prevalence of serotypes in this region. This study aims to figure out the prevalence of DENV serotypes in the samples collected from the dengue patients during the 2017 outbreaks in Peshawar, KP.

## 2 MATERIALS AND METHODS

### 2.1 Sample collection

The study was approved by the Ethics Committee of the IBGE, UAP. A total of 800 patients from various healthcare centers, showing DF symptoms during 2017 outbreak, were enrolled in the study. About 4 mL blood was collected from DF‐positive samples in EDTA tubes after the approval of informed consent from the selected patients. Collected blood samples were screened for the presence of dengue virus active infection according to standard biosafety protocol. The demographic and clinical history was recorded on a proforma.

### 2.2 Viral RNA isolation

The RNA was extracted from 150 µL serum sample using Favorgen viral RNA extraction kit (Favorgen Biotech Corp), following the manufacturer's instructions. Extracted RNA was either stored at −80°C or processed immediately. Method introduced by Lanciotti and colleagues in 1992 was used for single‐tube multiplex PCR, rapid detection, and typing of dengue virus in clinical samples.^18^

### 2.3 cDNA synthesis, amplification, and DENV serotyping

Four microlitre of extracted RNA was reverse transcribed with the help of Moloney‐murine leukemia virus (M‐MLV). PCR mix comprised of 4.0 μL 5× Buffer, 2.0 μL 5 pm outer antisense primer, 2.0 μL dNTPs (10 mmol/L), 1.0 μL MMLV (200 U/μL), and 1.0 μL dH_2_O to adjust the volume to 10 μL. The condition for RT‐PCR was as follows: initial denaturing for 42 minutes at 42°C, annealing at 60°C for 1 minutes, final temperature 92°C for 2 minutes, and holding temperature 22°C for 2 minutes. Two‐step PCR (1st and 2nd round) was carried out to amplify the synthesized cDNA and dengue virus serotyping. cDNA was amplified using forward and reverse primers: D1 represents the forward (sense) primers while D2 represents the reverse (antisense) primers. In the 2nd round of PCR, the reverse primers were replaced with four serotype‐specific primers. D1 and type specific 1 (TS1) were used for the identification of serotype 1, which amplify a region of 482 bp; D1 along with type specific 2 (TS2) was used to identify serotype 2, which amplify a region of 119 bp; and D1 and type specific 3 (TS3) amplified a region of 290 bp. Serotype 4 was amplified by D1 and type specific 4 (TS4), and a region of 392 bp was amplified. The primers used are shown in Table 1. PCR‐amplified products were detected by using agarose gel. 2% agarose gel was prepared in TBE buffer (1×), which was pre‐stained with 3 µL ethidium bromide that was used as a florescent dye. After electrophoresis, photographs were taken under ultraviolet light transilluminator. A 100 bp of DNA ladder was used as a DNA marker.

**Table 1:** Primers used for dengue virus serotyping Lanciotti et al^18^

Primer Name	Sequence (5′–3′)	bp amplified
D1 (Dengue Forward)	TCAATATGCTGAAACGCGCGAGAAACCG	511
D1 (Dengue Reverse)	TTGCACCAACAGTCAATGTCTTCAGGTTC	
TS1 (Type specific 1)	CGTCTCAGTGATCCGGGGG	482
TS2 (Type specific 2)	CGCCACAAGGGCCATGAACAG	119
TS3 (Type specific 3)	TAACATCATCATGAGACAGAGC	290
TS4 (Type specific 4)	CTCTGTTGTCTTAAACAAGAGA	392

## 3 RESULTS

**Figure 1:**
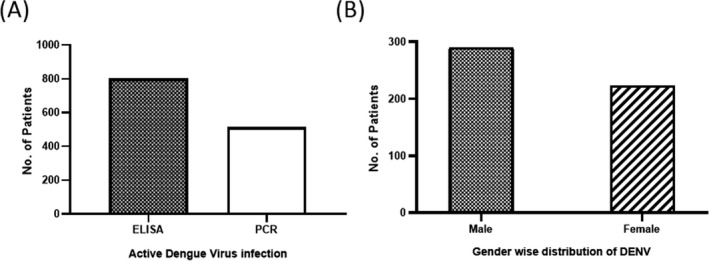
Active dengue virus infection. A, 800 samples were IgM/IgG positive, which was confirmed by ELISA (filled bar). All ELISA‐positive samples were further confirmed by RT‐PCR for active DENV infection (empty bar). B, Shows active infection in male and female

Patients with clinical manifestations of dengue and confirmed IgM (first antibodies to appear after primary DENV infection) or IgG‐positive samples were analyzed for the presence of active DENV. Initial screening was done for anti‐DENV using ELISA. Confirmation of active DENV infection was performed by RT‐PCR. Out of 800 collected samples, 513 (290 males, 223 females) samples (64.12%) were found to have active dengue virus (DENV) infection (Figure 1A).

**Figure 2:**
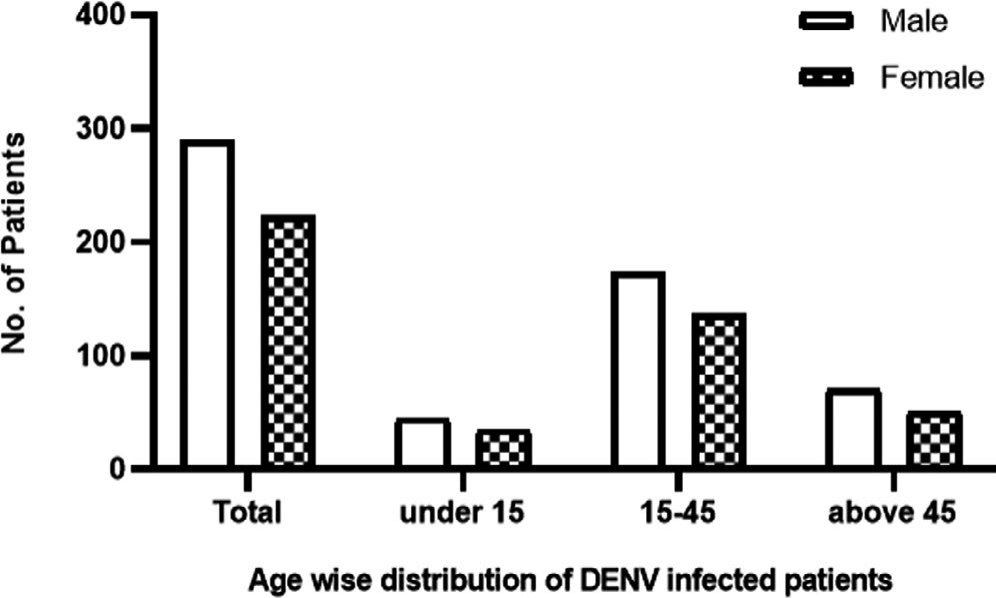
Age‐wise distribution of DENV‐infected patients. Empty bars represent the number of male patients belonging to different age groups, while bars with patterns show the female patients in three age groups

PCR‐positive patients were further analyzed for serotype‐specific detection with already reported primers.^18^ Among 513 PCR‐positive patients, 290 were males and 223 were females (Figure 1B). The patients were grouped into 3 classes according to age. First group was <15 years old; second group includes patients between 15 and 45 years old while the third group consisted of patients older than 45 years. Highest prevalence (60%) was found in the second group of patients with age from 15 to 45 years, while in case of younger age group (<15 years old) lowest DENV prevalence (15%) was recorded (Figure 2). Male:female ratio was quite similar in all age groups.

**Figure 3:**
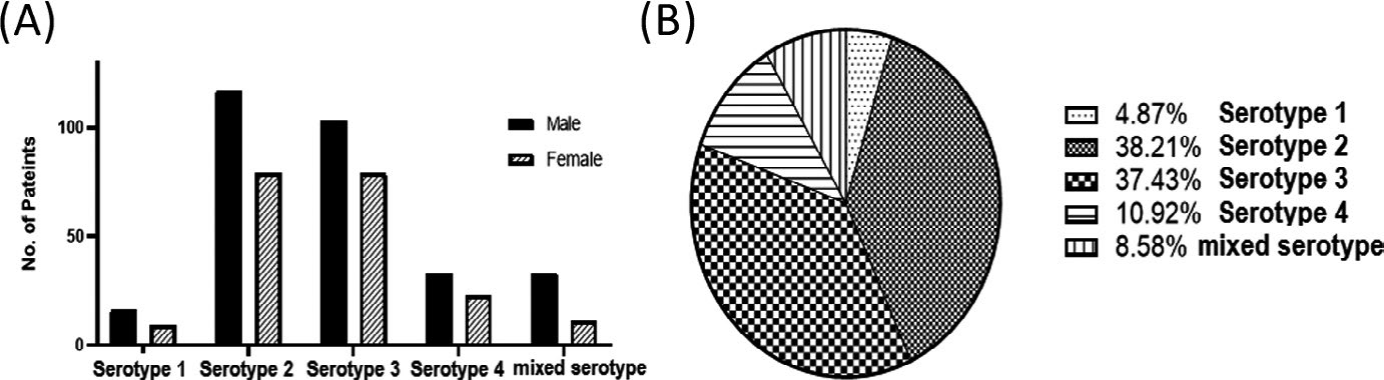
Serotype distribution of DENV in Peshawar, KPK. A, All DENV‐positive samples were further analyzed to identify the specific serotype. Bar graph represents the serotype distribution in male and female patients. B, Pie chart shows the percentage of all serotypes in DENV‐positive samples

Using serotype‐specific primers, specific serotypes were determined, as illustrated in Figure 3. Out of 513 patients, serotype 1 was the least prevalent serotype with only 4% cases, serotypes 2 and 3 were predominant serotypes accounting for 38% and 37%, respectively, whereas 10% positive DENV patients have serotype 4. Mixed circulating serotype was detected in approximately 9% patients.

## 4 DISCUSSION

Dengue is a fast emerging pandemic‐prone viral disease in many parts of the world. Dengue is caused by virus of Flaviviridae family, and there are 4 distinct, but closely related, serotypes that cause dengue (DENV‐1, DENV‐2, DENV‐3, and DENV‐4).^19^ The aim of our study is to investigate which DENV serotype was prevalent in the 2017 outbreak. DENV is first identified in 1982 in Pakistan^20^; however since 2006, dengue has become the most crucial vector‐borne disease in Pakistan. Once infected, humans become the main vectors and multipliers of the virus, acting as viral source for uninfected mosquitoes. At present, the main method to prevent the transmission of DENV is the control of mosquito populations.^2^ Over the past decade, the country has faced several outbreaks in which thousands of people have been infected and they even lost their lives.^4^, ^21^, ^22^, ^23^, ^24^, ^25^, ^26^, ^27^, ^28^ In Punjab, a populous province that shares a border with KP, in 2011 a serious epidemic affected over 50 000 people. In 2013, another huge outbreak of infection from KP province was reported with an estimated number of cases of 8546, including 33 deaths in the Swat district according to the WHO report. The prevalent serotypes were serotypes 1, 2, and 3.^29^, ^30^, ^31^, ^32^

The dengue virus may have been endemic to Pakistan, which remains unnoticed due to inadequate diagnostic and surveillance facilities. The majority of cases are asymptomatic or mild and self‐managed, so the actual number of dengue cases is underestimated. Many cases are also misdiagnosed as other febrile illnesses.

Dengue infection is very common among travelers from tropical and subtropical regions. The risk of infection in travelers usually depends on exposure to risk factors and the local prevalence of the infection. No prophylaxis is available to prevent dengue. On the other hand, nosocomial transmission may occur in areas where dengue is endemic. This may be especially relevant to healthcare workers who care for patients with dengue with hemorrhage.^33^

Viral transmission without a mosquito vector has been rarely reported. The routes of transmission include needlestick injuries, bone marrow transplantation, and intrapartum and vertical transmission.

The KP population is exposed to all kinds of deadly diseases because the etiological factors for most diseases are found mainly in this province. The country has been battered by the war, the last one has been fought against terrorism, and therefore, there have been outbreaks of many deadly diseases. However, many diseases have been controlled and their spread has been overcome, but adequate planning is needed to combat a deadly disease. Efficient strategy should not only neutralize the viral spread but should also include eliminating or reducing risk factors. Although the province's health system is constantly improving, there are many problems to be solved.

The Ministry of Health in Pakistan has taken several response measures, such as vector surveillance and control activities have been initiated, and all provinces have arranged free‐of‐charge diagnostic and clinical management services for the cases.^34^

Efficient medical assistance can limit the progression of the disease and can save lives, reducing mortality rates from more than 20% to <1%.^2^ Contaminated environment contributes to the onset of viral outbreaks. During the epidemic of July 19, 2017, dengue affected 95 000 people. According to the World Health Organization, in Peshawar, the capital KP province of KP, the largest number of cases was reported with a total of 88 000 suspected cases, including 19 000 confirmed cases and 56 deaths (CFR 0,3%) on October 24, 2017.

The first step to solving a problem is to quantify and identify its nature. Similarly to combat the virus, we first need to find out what its actual prevalence is in a particular area. In particular, in our study eight hundred ELISA‐positive samples were collected, of which 513 (290 males, 223 females) samples were confirmed PCR positive. The highest prevalence was observed in the age group 15‐45 years with 308 patients (60%). Out of 513, 25 had serotype 1 (16 males, 9 females), 196 had serotype 2 (117 males, 79 females), 192 had serotype 3 (103 males, 79 females), 56 had serotype 4 (33 males, 23 females), and mixed serotypes were found in 44 (33 males, 11 females). Hence, we conclude that serotypes 2 and 3 were the prevalent serotypes (38% and 37%, respectively) of dengue in the 2017s outbreak. Our results are in accordance with previous reports suggesting that the age group 15‐45 years is typical of infection in the area, and the serotypes 2 and 3 remain the most common in previous outbreaks in Pakistan as summarized in Table 2. Various studies published in the last decade have reported DENV‐2 and DENV‐3 as predominant serotype except Humayoun and colleagues, which found DENV 4 as prevalent serotype.^4^, ^21^, ^22^, ^23^, ^24^, ^25^, ^26^, ^27^, ^28^

**Table 2:** Most prevalent DENV serotypes during last decade (2008‐2018) in Pakistan

Year of outbreak	Predominant Serotype	Location	References
2008	DENV‐4	Lahore, Punjab	Humayoun et al^4^
2007‐2009	DENV‐2	Lahore	Fatima et al^21^
2010	DENV‐2 and DENV‐3	Lahore, Sheikhpura, and Gujranwala	Mahmood^22^
2011‐2013	DENV‐2 and DENV‐3	Punjab Swat, KP	Ali et al^23^
2011	DENV‐2	Lahore	Idrees et al^24^
2011	DENV‐2	Lahore	Khan et al^25^
2013	DENV‐2 and DENV‐3	Swat, KP	Ali et al^26^
2013	DENV2	Rawalpindi, Lahore, Faisalabad, Multan, Swat	Shahid et al^27^
2013	DENV‐3	Swat, KP	Ali et al^26^
2013	DENV‐2 and DENV‐3	Swat	Khan, Ghaffar, & Khan^28^
2017	DENV2	Peshawar, KP	This study

Dengue flourishes in urban poor areas, in the suburbs and in the countryside, but it also affects the more affluent neighborhoods in countries located in the tropics and subtropics.

In Pakistan, people are exposed to comorbidities, infected blood products, and many risk factors. The risk factors are influenced by local spatial variations of rainfall, temperature, relative humidity, degree of urbanization, and quality of vector control services in urban areas.

The recommendation to combat the disease is to inform the local community about risk factors and potentially fatal complications of dengue virus. Awareness seminars and workshops should be held to educate the population. In particular, healthcare workers should be employed for the cause so that they can carry out a massive campaign and inform people to take preventive measures to combat dengue.

In conclusion, the incidence of dengue has grown dramatically in the last decade in Pakistan. In our study, serotypes 2 and 3 were the predominated serotypes of dengue virus in the 2017 outbreak in Peshawar. To prevent and combat the spread of dengue is necessary an efficient monitoring and evaluation strategy. This includes the disease surveillance through the identification and molecular characterization of infected human cases, and the vector surveillance by tracking mosquito populations in areas of potential. In addition, monitoring of behavioral impact is essential for observing whether the strategies aimed to reduce dengue transmission are adopted and supported by the community.

## CONFLICT OF INTEREST

The authors declare that there is no conflict of interests regarding the publication of this paper.

## ETHICAL APPROVAL

The Ethics Committee of the IBGE, UAP, granted approval for the study that was conducted according to the principles of ICH‐GCP and Declaration of Helsinki.
